# The Effect of Mandala Colouring on Spiritual and Psychological Well‐Being in Family Members Caring for Palliative Care Patients: Experimental Study

**DOI:** 10.1111/scs.70235

**Published:** 2026-04-02

**Authors:** Arzu Nurdaş

**Affiliations:** ^1^ Department of Nursing Kyrenia University Faculty of Health Sciences Kyrenia Cyprus

**Keywords:** mandala, palliative, psychological well‐being, spiritual well‐being, well‐beingdeath

## Abstract

**Background:**

Palliative care is a broad spectrum of care that encompasses not only the physical but also the psychological and spiritual care of patients at the end of life, as well as the care of family members caring for the patient.

**Objective:**

The aim of the study was to evaluate the effect of mandala colouring on spiritual and psychological well‐being in family members caring for palliative care patients.

**Material and Method:**

A total of 84 relatives of palliative care patients (42 experimental and 42 control) participated in this prospective, randomised, controlled, experimental study. The relatives of palliative care patients in the experimental group were given mandala colouring for 4 weeks, once a week for 1 h. No intervention was made to the relatives of the patients receiving palliative care services in the control group. Mandala colouring book and crayon were used for the study. The application was done quietly, calmly, and outside the clinic area.

**Data Collection and Analysis:**

Data were collected with personal information form, Psychological Well‐Being scale, and Spiritual Well‐Being scale. Data were analysed using SPSS 26 software.

**Main Results:**

When the pre and post‐test results obtained from the experimental group were evaluated, the level of well‐being increased, and as a result, mandala colouring was effective in increasing spiritual and psychological well‐being.

## 1 Introduction

Mortality is a common and universal condition that we humans and all living organisms share. Nevertheless, the attitudes and behaviours of people towards death are quite different. After the death of the patient, the rest of the people go through a mourning process. Sometimes this mourning process may begin in the terminal period before the patient is lost. Palliative care is a wide‐ranging form of care that covers the physical as well as psychological and spiritual care of patients at the end of life and similarly includes the care of family members who care for the patient [1, 2, 3].

The concept of care provided to palliative care patients is the task of undertaking all the activities carried out for the purpose of care and the responsibilities of the patient. This multifaceted concept of care includes physical care as well as emotional, spiritual, social and economic aspects [4, 5, 6]. This approach is a holistic approach that covers mental, sociocultural and spiritual dimensions as well as physical needs. The psychological and spiritual health of the patient's relatives is as important as that of the patient [6, 7]. The multidimensional concept of psychological well‐being, which includes many different factors, includes the individual's positive and negative affect, satisfaction with life, and satisfaction with life together with the individual's well‐being [8]. Similarly, spiritual well‐being, which carries a religious and existential dimension, increases the energy and motivation to cope with painful feelings such as loneliness, depression, and loss that arise when an individual experiences any tension [9]. There are studies showing that approach‐oriented coping methods are positively effective on well‐being [10]. Similarly, mindfulness and art‐based practices remain up to date [11]. People of almost every age and culture who see art as a language go into the unconscious to heal their pain and use it in situations such as psychological strengthening or compensating for loss [12, 13].

In the art therapy intervention, themes such as fear of death or illness, loneliness and the finitude of existence were dealt with non‐verbally. The benefit of this approach is that problems are recognised and dealt with in a rational and defensive manner. Works of art represent the embodiment of emotions and are used to gain insight into problems. Change, which is the main goal of psychotherapy, can enable the individual to take a shape in art therapy. An impressive universe, different from the daily routine, can create new perceptions [14]. Ploukou and Panagopoulou [15] observed that making improvised music with percussion instruments improves depression, anxiety and psychological distress [15]. Kim et al. [16] observed that art‐based mandala practice increased subjective well‐being [16].

Mandala, one of the art‐based practices, includes shapes, ornaments or rituals used in different religions and beliefs. Mandala literally means “circle”, “circle”. Again, in some translations, it is seen that it is sometimes used in the meaning of “centre” and sometimes “surrounding thing” [17]. According to Carl Jung, the founder of analytical psychology, the drawing of a circle with a centre is an allegory about the nature of God known since ancient times [18]. Mandalas, in their simplest form, are circles in a square, all arranged in sections around a single central point. They are generally made on paper or fabric. Although mandalas give extraordinary images as independent works of art, they have a symbolic and meditative meaning beyond this appearance [19]. During the instant expression with mandala, the individual uses colours that come from within and derives symbols [20].

Attitudes developed towards the death and dying process, which is a natural requirement of existence, may differ depending on individual beliefs. Individuals with high spiritual and psychological well‐being can be more hopeful, successful, and productive by making sense of their lives [8, 21]. In the palliative care process, it is possible that individuals who care for the patient as well as the patient are under stress and experience feelings that reduce well‐being, such as loneliness, depression, and isolation. The difficulties and burden of care experienced by family members caring for palliative care patients are mentioned in the literature [22, 23, 24]. For all these reasons, palliative care service also includes the support of patient relatives who are about to lose or have lost their patient [25]. Although different studies have been conducted on the problems experienced by the relatives of palliative care patients, no study has been found to examine the effect of mandala colouring on the spiritual and psychological well‐being of the relatives of palliative care patients. Based on this deficiency, the study was designed to examine the effect of mandala colouring on spiritual and psychological well‐being in the caregivers of palliative care patients.

## 2 Method

The study was prospective, randomised, controlled, and experimental. The aim of the study is to determine whether mandala colouring is effective on the well‐being of patients' relatives in palliative care and to guide research.

### 2.1 Participants

The population of the study consisted of family members caring for their patients in the palliative clinic of İstanbul Çatalca State Hospital. The sample was determined as all volunteers who met the inclusion criteria during the study period (between 25 August and 25 November 2022). G*Power 3.1 package programme was used for the power calculation of the study [26]. As a result of the post hoc power analysis (Cohen's *d* = , *f* = , *η* ^2^ = ) the sample size was found to be sufficient. A post hoc power analysis revealed 100% power to detect a large effect of 2.86.

The study period was 3 months and was planned between 25 August and 25 November 2022. Companions of patients hospitalised for at least 1 month were included in the study.

### 2.2 Inclusion Criteria

Being a patient caregiver in the palliative clinic where the research was conducted. Not using antidepressants and anxiolytics. No physical disability that will prevent mandala colouring. Not being allergic to mandala colouring materials. To be 18 years of age or older. Not having any communication problems. Relatives of patients who agreed to participate in the study were included in the study.

A total of 84 relatives of patients receiving palliative care services, 42 experimental and 42 control, participated in the prospective, randomised controlled, experimental type study. Using block randomization, 84 participants were divided into 4‐person blocks (AABB, ABAB, ABBA, BAAB, BABA, BBAA) into groups A (experimental group) and B (control group). The relatives of palliative care patients in the experimental group were given mandala colouring for 4 weeks, once a week for 1 h. No intervention was made to the relatives of the patients receiving palliative care services in the control group (Consort Flow).

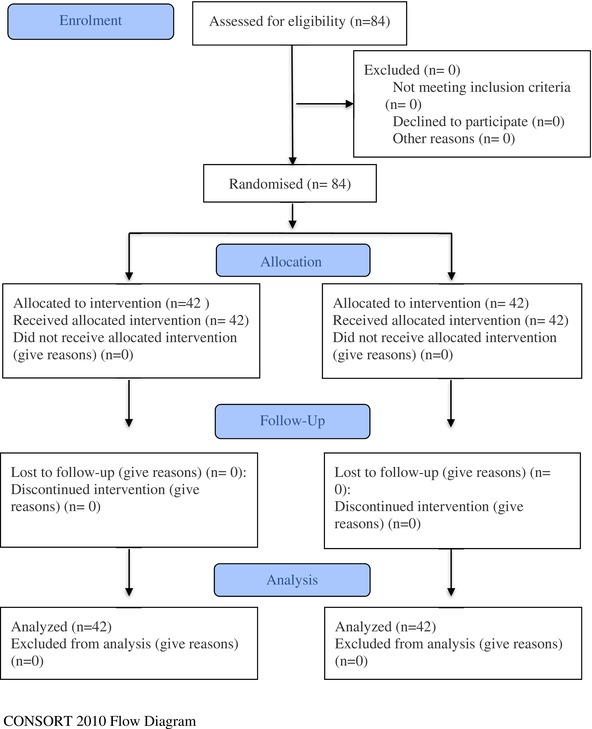

**FIGURE 1:**
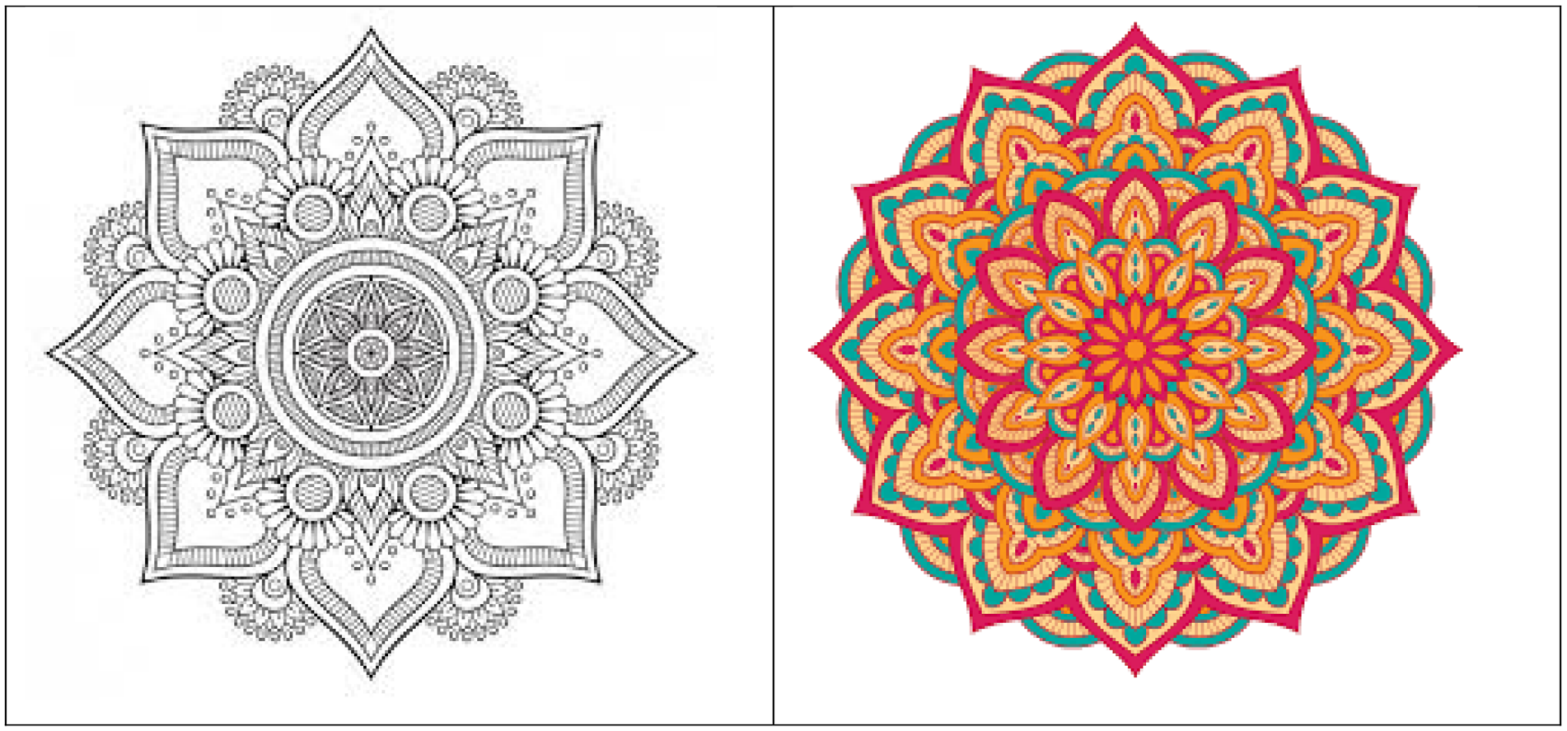
Mandala pattern. Mandala pattern (https://depositphotos.com/tr/serie/382932328.html).

Work pattern for Figure 1: Participants came to the meeting room just outside the hospital clinic once a week (on Mondays) for 4 weeks. Participants were given mandala‐patterned paper and coloured pencils. Participants were provided with a quiet environment. They were instructed to turn off their phones, refrain from talking to each other as much as possible, and concentrate on colouring the mandalas. This activity lasted 1 h. The researcher was present in the room throughout the activity but did not intervene. The patients the participants were responsible for looking after were left under the supervision of a nurse in the clinic for 1 h and did not participate in the activity.

### 2.3 Ethics Approval

The study was conducted after the approval of the ethics committee dated 28.07.2022 and numbered 2022.07.178 from Istanbul Kanuni Sultan Süleyman Education and Research Hospital. The study was conducted in accordance with the Declaration of Helsinki. Consent was obtained from participants. The participants completed an information form and a scale as pretest and post‐test. All data were collected anonymously and confidentially.

### 2.4 Data Collection Tools

Data were collected with personal information form, Psychological Well‐Being scale and Spiritual Well‐Being scale. The Spiritual Well‐Being scale developed by Eksi and Kardas consists of transcendence, harmony with nature and anomie sub‐dimensions. Each item ranges from 1 to 5; as the total score increases, spiritual well‐being increases. The total Cronbach's alpha value of the scale is 0.89. In our study, the Cronbach's alpha values of the Spiritual Well‐Being Scale were 0.94, 0.79 and.87 for transcendence, harmony with nature and anomie sub‐dimensions, respectively [27].

The Psychological Well‐Being Scale was developed by Diener et al. [28] and includes eight statements; the scores of the scale vary between 8 and 56, and the higher the score, the higher the psychological well‐being. In the validity and reliability study conducted by Telef in 2013, the Cronbach alpha internal consistency coefficient of the scale was found to be 0.87. The Cronbach's alpha coefficient of our study was 0.83 [29].

### 2.5 Data Collection Process

All participants completed the data collection tools before starting the study. Relatives of palliative care patients in the experimental group were given mandala colouring for 4 weeks, once a week for 1 h. No intervention was made to the relatives of the patients receiving palliative care services in the control group. At the end of the study at the end of 4 weeks, the data collection tools were filled in again.

### 2.6 Statistical Analysis

Data collection tools were administered to both experimental and control groups at the beginning of the study and 4 weeks later. The data were analysed using SPSS 26 software. The chi‐square test of independence was used to examine whether there were statistically significant differences in the distribution of gender, marital status, childbearing status, educational level, economic situation, and degree of proximity to the patient between the experimental and control groups. In addition, independent sample *t*‐tests were conducted to determine whether there were statistically significant differences between the groups in terms of the mean scores for age, number of people responsible for patient care, and number of days of care provided per month.

**FIGURE 2:**
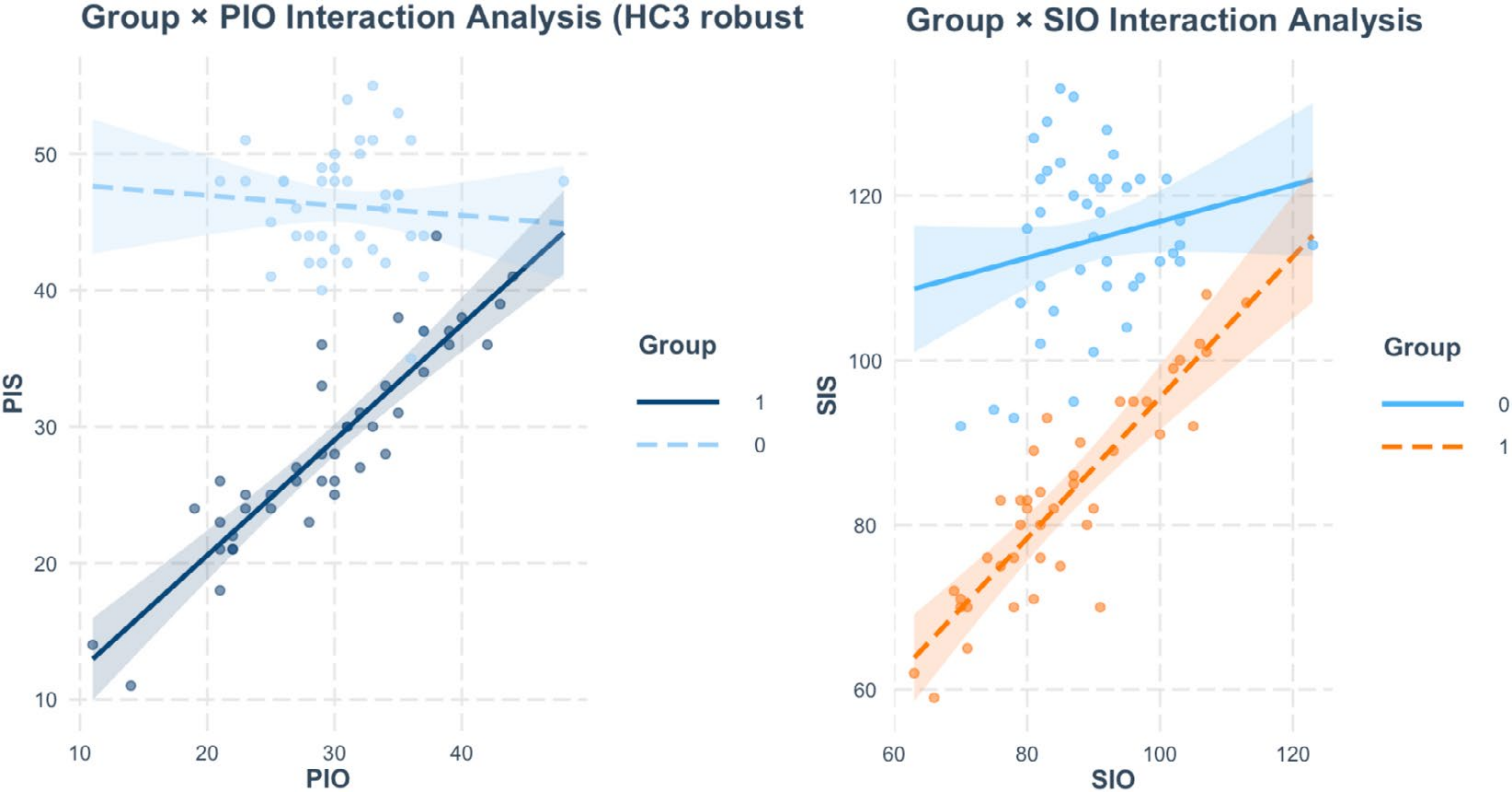
The group × PIO and group × SIO interaction interactions, illustrating the heterogeneity of regression slopes.

We first ran an ANCOVA with the pretest scores as covariates to examine experimental effect; however, the homogeneity‐of‐slopes assumption was violated for both spiritual and psychological well‐being: the Group × pre‐SIO (*F*_1,80_ = 13.82, *p* < 0.001) and Group × pre‐PIO (*F*_1,80_ = 41.90, *p* < 0.001) interactions were significant (see Figure 2). These violations imply that the regression slopes relating the covariate (pretest) to the outcomes (SIS and PIS) differ between groups, that is, the regression lines are not parallel. Consequently, we turned to a difference‐in‐differences (DiD) analysis. DiD is a quasi‐experimental approach widely used in estimate causal effects; it requires data for two groups—one treated and one untreated—observed at two points in time (pre‐ and post‐intervention) and rests on the assumption that, in the absence of treatment, the two groups would have followed parallel trends [30].

## 3 Results

The results regarding the descriptive characteristics of the participants and the results of the DiD analysis are presented below, respectively.

### 3.1 Descriptive Characteristics of the Cases

The distribution of the descriptive characteristics of the subjects is given in Table 1. As seen in the table, the majority of both the experimental and control groups were female and married; the mean age was 45.6 years in the experimental group and 46.2 years in the control group. While 33.6% of the subjects in the experimental group had children, this rate was 19% in the control group. In terms of educational status, those who graduated from primary school were more in the experimental group (57.1%) and those who graduated from high school were more in the control group (71.4%), and there was a statistically significant difference between the two groups in terms of educational status (*p* < 0.001). More than half of the participants in the experimental group defined their economic status as poor, while more than half of the participants in the control group defined it as moderate.

**TABLE 1:** Descriptive characteristics of the cases (*N* = 84).

Descriptive characteristics	Experiment (*n* = 42)	Control (*n* = 42)	Statistical test, significance
	*n* (%)	*n* (%)	
Gender			
Woman	27 (64.3)	30 (71.4)	*χ* ^2^: 0.64
Man	15 (35.7)	12 (28.6)	*p*: 0.64
Marital status			
Single	12 (28.6)	7 (16.7)	*χ* ^2^: 1.70
Married	30 (71.4)	35 (83.3)	*p*: 0.19
Childbearing status			
There is	14 (33.6)	8 (19.0)	*χ* ^2^: 2.22
None	28 (66.7)	34 (81.0)	*p*: 0.24
Education status			
Primary education	24 (57.1)	1 (2.4)	*χ* ^2^: 37.20
High School	6 (14.3)	30 (71.4)	*p*: 0.00**
University	12 (28.6)	11 (26.2)	
Economic situation			
Medium	17 (40.5)	23 (54.8)	*χ* ^2^: 1.72
Bad	25 (59.5)	19 (45.2)	*p*: 0.28
Degree of proximity to the patient			
Partner	14 (33.3)	9 (21.4)	*χ* ^2^: 3.36
Son/daughter	17 (40.5)	22 (52.4)	*p*: 0.34
Relative	4 (9.5)	7 (16.7)	
Carer	7 (16.7)	4 (9.5)	
Age (mean ± ss years)	45.64 ± 11.40	46.19 ± 11.24	*t*: 0.22
Min‐max	22–68	26–68	*p*: 0.83
Number of people responsible for patient care (mean ± ss years)	1.69 ± 0.56	2.16 ± 1.20	*t*: 2.32
Min‐max	1–3	1–8	*p*: 0.02*
Number of days of care provided/month (mean ± ss years)	19.92 ± 9.04	17.47 ± 8.87	*t*: 1.25
Min‐max	5–30	5–30	*p*: 0.21

*Note:* *t* = *t*‐test (independent groups); chi‐square test; *p < 0.05; **p < 0.001.

In both groups, the majority of the caregivers were the son/daughter of the patient. The mean number of people responsible for patient care was 1.69 in the experimental group and 2.16 in the control group, and there was a statistically significant difference between the two groups (*p* < 0.05). Those in the experimental group have been caring for the patient for approximately 20 days, while those in the control group have been caring for the patient for 17 days.

The experimental (*n* = 42) and control (*n* = 42) groups were similar in terms of sex distribution, marital status, childbearing status, economic situation, and degree of proximity to the patient (all *p* > 0.05). The mean age (*p* = 0.83) and number of care days per month (*p* = 0.21) did not differ significantly between the groups.

Education level differed significantly between groups, *χ* ^2^ = 37.20, *p* < 0.001, with the experimental group having more participants with primary education and the control group having more with high school education. However, the number of people responsible for patient care was significantly higher in the control group (*M* = 2.16, SD = 1.20) than in the experimental group (*M* = 1.69, SD = 0.56), *t* = 2.32, *p* = 0.02.

The mean scores on the pretest measures suggest that the two groups were similar at baseline, particularly on the Psychological Well‐Being Pretest (PIO: *M*_Experiment_ = 31.10 vs. *M*_Control_ = 29.38). On the Spiritual Well‐Being Pretest (SIO), the experimental group had a slightly higher mean (*M*_Experiment_ = 89.93 vs. *M*_Control_ = 85.62). The standard deviations indicate that the Control group generally showed greater variability on the pretest scores (SIO: SD = 12.55 vs. 9.52; PIO: SD = 7.72 vs. 4.90), suggesting a wider spread of scores compared to the Experiment group before intervention in Table 2.

**TABLE 2:** Group descriptive statistics.

Group	*n*	SIO *M* (SD)	PIO *M* (SD)	SIS *M* (SD)	PIS *M* (SD)
Experimental	39	89.93 (9.52)	31.10 (4.90)	114.64 (10.37)	46.14 (4.34)
Control	40	85.62 (12.55)	29.38 (7.72)	83.19 (12.07)	28.50 (7.18)

Abbreviations: PIO, Psychological Well‐Being Pretest; PIS, Psychological Well‐Being Posttest; SIO, Spiritual Well‐Being Pretest; SIS, Spiritual Well‐Being Posttest.

Following the intervention, the experimental group demonstrated a substantially higher mean score on the Spiritual Well‐Being Posttest (SIS: *M* = 114.64) than did the Control Group (*M* = 83.19). This large difference suggests a strong positive effect of the intervention on spiritual well‐being. Similarly, the experimental group reported a much higher mean score on the Psychological Well‐Being Posttest (PIS: *M* = 46.14) than did the Control Group (*M* = 28.50). This also suggests a considerable positive impact of the experimental intervention in Table 2.

The descriptive data indicated that while the two groups were relatively comparable at baseline (pretest), the experimental group showed markedly higher means and apparent gains on both Spiritual Well‐Being and Psychological Well‐Being measures post‐intervention. Since the Homogeneity of Regression Slopes assumption was not met for both tests, the data were analysed using the difference in difference (DiD) instead of ANCOVA, and the results are presented in Table 3.

**TABLE 3:** Results of difference in difference test for spiritual and Psychological Well‐Being Scores.

Outcome	Term	Estimate	SE	*p*	Conf. Low	Conf. High
Spiritual	ATT (2,2)	27.14	2.03	0.00***	22.97	31.32
Psychological	ATT (2,2)	15.93	1.20	0.00***	13.69	18.17

*Note:* The * indicates significance levels: ***p < 0.001.

Abbreviation: ATT, Average Treatment Effect on the Treated.

The intervention yielded statistically significant improvements in both outcome measures (Table 3). Regarding spiritual well‐being, the Average Treatment Effect on the Treated (ATT) was 27.14 points (SE = 2.03, *p* < 0.001, 95% CI [22.97, 31.32]), reflecting a pre‐to post‐test mean difference of 24.71 points in the experimental group compared to a decline of 2.43 points in the control group. For psychological wellbeing, the ATT reached 15.93 points (SE = 1.20, *p* < 0.001, 95% CI [13.69, 18.17]), with the experimental group demonstrating a 15.04‐point improvement from baseline while the control group experienced a modest decrease of 0.88 points. The magnitude of these effects, combined with the narrow confidence intervals and highly significant *p*‐values, suggests that the intervention produced clinically meaningful improvements in both spiritual and psychological well‐being outcomes among the treated participants.

**FIGURE 3:**
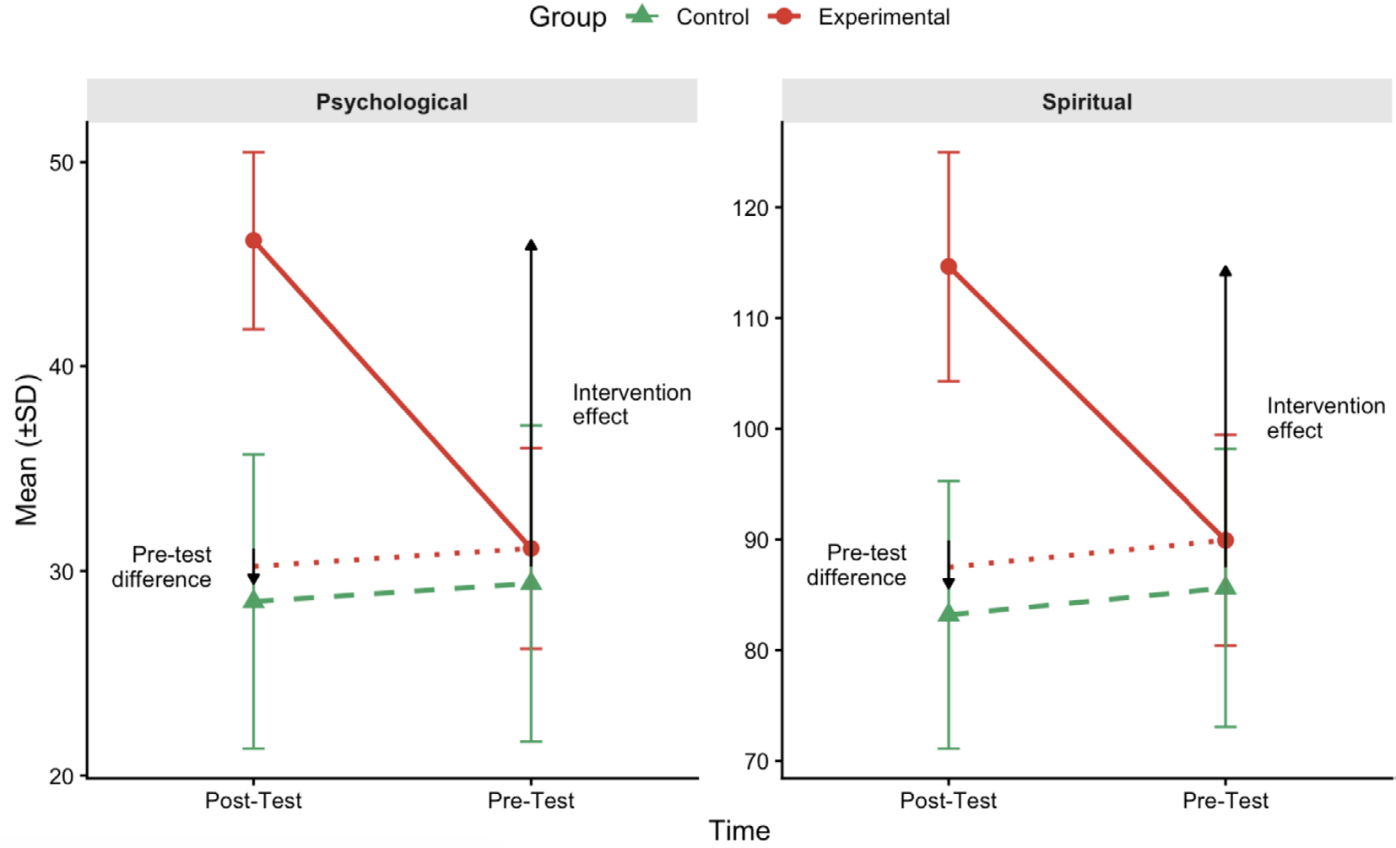
Difference‐in‐differences analysis: visual representation of treatment effects on spiritual and psychological well‐being.

Figure 3 illustrates the DiD analysis comparing the experimental and control groups on spiritual and psychological well‐being from pretest to post‐test. For spiritual well‐being, the experimental group (solid line) showed a substantial increase from the pretest (*M*≈89.9, SD≈9.5) to post‐test (*M*≈114.6, SD≈10.4), whereas the control group (dashed line) remained relatively stable from the pretest (*M*≈85.6, SD≈12.6) to post‐test (*M*≈83.2, SD≈12.1). For psychological well‐being, the experimental group (solid line) also improved notably from pretest (*M*≈31.1, SD≈4.9) to post‐test (*M*≈46.1, SD≈4.3), while the control group (dashed line) showed minimal change from pretest (*M*≈29.4, SD≈7.7) to post‐test (*M*≈28.5, SD≈7.2). Error bars represent ±1 standard deviation. The dotted lines represent the counterfactual trajectories—what the experimental group's post‐test scores would have been in the absence of intervention. The vertical arrows labelled “Intervention effect” indicate the magnitude of the treatment effect, calculated as the difference between the observed post‐test scores (solid line) and counterfactual estimates (dotted line). The arrows labelled “Pretest difference” show the baseline differences between the groups before the intervention. Assuming parallel trends between the experimental and control groups prior to the intervention, the divergence between the observed post‐test outcomes and the estimated counterfactual trajectories suggests a positive intervention effect on both spiritual and psychological well‐being.

## 4 Discussion

Palliative care encompasses psychological and spiritual care as well as physical care of the patient and supports the family members caring for the patient. In the present study examining the Effect of Mandala Colouring on Spiritual and Psychological Well‐Being in Family Members Caring for Palliative Care Patients, it was observed that mandala colouring in the experimental group increased both spiritual and psychological well‐being mean scores with statistical significance.

Access to the arts for the care provided to palliative care patients can support mental and physical health and emotional well‐being. Vaartio‐Rajalin et al. [31] emphasised that art activities should be a fundamental part of nursing care in their review of 42 articles supporting this view. Because art activities have been found useful in every context such as prevention, rehabilitation, care, treatment and palliation [31].

Art enables people in difficult situations to embody their feelings and gain insight into problems [14]. Previous studies have shown that art (music, dance and visual art therapy) intervention for family caregivers has a positive effect on criteria such as burden, depression, self‐efficacy, and social and mental well‐being [32, 33].

Mandala, one of the visual arts, is also a therapy method due to its ability to provide expression and transfer. Kim et al. [16] observed that mandala colouring increased subjective well‐being [16]. In their study, Liu et al. [34] found that mandala reduces negative emotions, however, it is beneficial to increase the spiritual level of the individual and significantly improves spirituality.

The present study showed that mandala colouring has a positive effect on psychological well‐being. The literature suggests that mandala colouring eliminates problems such as stress [35, 36, 37], anxiety [38, 39, 40, 41], and negative emotions [34] that affect people's well‐being. Meutia [35] showed that mandalas are effective on perceived stress. 32 adult participants participated in the experimental study and painted a structured mandala three times and were asked to fill out the Perceived Stress Scale before and after mandala painting, and the data showed that the mandala was effective on perceived stress [35]. Similarly, in the McDougall study [36], in order to examine the effect of mandalas on stress, after a stressful task given to the participants through an online survey platform, mandala painting, free drawing, and a video of someone watching a visual artwork were shown. It showed that the effect of mandala cannot be obtained by watching remotely [36]. In another study on stress, the effectiveness of mandala painting paired with breathing exercise in reducing negative affect, state anxiety, and psychophysiological stress response following a given stressor (Trier Social Stress Test) was measured; a significant decrease in negative affect and a tendency towards a significant decrease in state anxiety were found. While evaluating the psychophysiological stress response, blood pressure, pulse rate, skin conductance levels, and heart rate were measured throughout the experiment; it was stated that more similar studies are needed [37].

Looking at previous studies on pain and anxiety experienced in physiological disorders, mandalas were found to be more effective than sudoku on the anxiety of breast cancer patients receiving chemotherapy treatment, and in another study, it was found to reduce the anxiety and pain experienced by patients with burn dressings [38, 41].

Thanks to the innovation of the mandala over time and its applicability in digital environments; the effect of mandala colouring with “Pigmentation”, an augmented reality colouring software, on state awareness and dispositional flow was examined with 76 students at a university in China and it was shown that there was no effect on the state of awareness, but there was an improvement in the flow state [42].

Based on the current study and in the light of other studies, it was concluded that mandala colouring is an effective method. In addition to all the positive results, mandala colouring is not costly and does not require a workshop or special training.

## 5 Limitations

This study has certain limitations. Firstly, the fact that the research was conducted in a single clinical setting limits the generalisability of the findings to different clinical populations and cultural contexts. Therefore, it is thought that future studies conducted in different institutions and with groups with various sample characteristics will increase the external validity of the findings.

Another limitation is that the effects of the mandala study were largely assessed through self‐report scales. This may have led to measurement errors such as social desirability bias and the possibility of not fully reflecting the participants' emotional states. In future studies, the use of physiological measurements (e.g., heart rate, cortisol levels) or observation‐based assessment methods, in addition to self‐report data, may increase the validity of the findings.

## 6 Conclusion

As a result, mandala colouring was effective in increasing spiritual and psychological well‐being in family members of palliative patients. Many studies in the literature have revealed that carer well‐being, patient interaction, and patient care quality are parallel to each other [4]. Based on the findings of the current study and studies in the literature, it can be said that such interventions are needed.

Future studies should focus on multi‐centre research designs in order to examine the effects of mandala painting in greater depth. It is important that these studies are conducted using larger samples and combining different evaluation methods. Such studies can provide stronger evidence regarding both the effectiveness and sustainability of the intervention.

## 7 Recommendations

More frequent implementation of artistic activities related to patient relatives.

Providing the environment and materials for the mandala colouring work and encouraging the relatives of the patients for the work.

Training a person who can provide guidance on mandala colouring.

It is suggested to create curiosity and encouragement by hanging the mandala colouring works in patient rooms and clinic corridors.

## Author Contributions

Concept – A.N. Design – A.N. Supervision – A.N. Resources – A.N. Materials – A.N. Data collection and/or processing – A.N. Analysis and/or interpretation – A.N. Literature search – A.N. Writing manuscript – A.N. Critical review – A.N. Other – A.N.

## Funding

The author has nothing to report.

## Ethics Statement

The study was conducted after the approval of the ethics committee dated 28.07.2022 and numbered 2022.07.178 from Istanbul Kanuni Sultan Süleyman Education and Research Hospital. The study was conducted in accordance with the Declaration of Helsinki. Consent was obtained from participants. The participants completed an information form and a scale as pretest and posttest. All data were collected anonymously and confidentially.

## Consent

Informed consent was obtained from the participants and recorded.

## Conflicts of Interest

The author declares no conflicts of interest.

## Data Availability

The datasets generated and/or analysed during this study are not publicly available due to ethical and privacy considerations, as they involve sensitive data from relatives of patients receiving palliative care. The study was conducted in accordance with the Declaration of Helsinki and approved by the Institutional Ethics Committee (Approval No: 2022.07.178, 28 July 2022). In accordance with ethical approval conditions and to protect participant confidentiality, the data cannot be shared publicly. However, anonymised data are available from the corresponding author upon reasonable request and with permission from the relevant ethics committee.
